# Individualized social niches in animals: Theoretical clarifications and processes of niche change

**DOI:** 10.1093/biosci/biad122

**Published:** 2024-02-07

**Authors:** Marie I Kaiser, Jürgen Gadau, Sylvia Kaiser, Caroline Müller, S Helene Richter

**Affiliations:** Department of Philosophy, Bielefeld University, Bielefeld, Germany; Joint Institute for Individualisation in a Changing Environment, University of Münster and Bielefeld University, Münster and in Bielefeld, Germany; Institute for Evolution and Biodiversity, University of Münster, Münster, Germany; Joint Institute for Individualisation in a Changing Environment, University of Münster and Bielefeld University, Münster and in Bielefeld, Germany; Department of Behavioural Biology, Bielefeld University, Bielefeld, Germany; Joint Institute for Individualisation in a Changing Environment, University of Münster and Bielefeld University, Münster and in Bielefeld, Germany; Department of Chemical Ecology, Bielefeld University, Bielefeld, Germany; Joint Institute for Individualisation in a Changing Environment, University of Münster and Bielefeld University, Münster and in Bielefeld, Germany; Department of Behavioural Biology, Bielefeld University, Bielefeld, Germany; Joint Institute for Individualisation in a Changing Environment, University of Münster and Bielefeld University, Münster and in Bielefeld, Germany

**Keywords:** social niche, individualized niche, social environment, social interactions, social role, niche construction, niche conformance, niche choice, social niche specialization

## Abstract

What are social niches, and how do they arise and change? Our first goal in the present article is to clarify the concept of an individualized social niche and to distinguish it from related concepts, such as a social environment and a social role. We argue that focal individuals are integral parts of individualized social niches and that social interactions with conspecifics are further core elements of social niches. Our second goal in the present article is to characterize three types of processes—social niche construction, conformance, and choice (social NC^3^ processes)—that explain how individualized social niches originate and change. Our approach brings together studies of behavior, ecology, and evolution and integrates social niches into the broader concept of an individualized ecological niche. We show how clarifying the concept of a social niche and recognizing the differences between the three social NC^3^ processes enhance and stimulate empirical research.

The niche concept is central to ecological research and has recently gained new prominence in evolutionary and behavioral biology. Because individuals differ in their phenotypes and interact differently with their environment, ecological niches should be studied not only at the species or population level but also at the level of the individual. An individualized niche can be understood as the subset of the species’s niche that arises from the interactions of one (focal) individual with its environment (https://doi.org/10.32942/osf.io/7h5xq [preprint: not peer reviewed]). Like Hutchinson's niche concept, which is focused on species or populations (Hutchinson 1957), the individualized niche is an *n*-dimensional space that includes those abiotic and biotic environmental conditions that influence the fitness of an individual (Müller et al. 2020, Trappes 2021, Trappes et al. 2022).

Therefore, to understand how individualized niches originate and change, and which ecological and evolutionary consequences they have, an individual's interactions with both the abiotic and the biotic environment need to be considered. A significant subset of the biotic environment are conspecifics. In this context, many researchers use the concept of a social niche to emphasize that they investigate, for instance, which social roles individuals play, in which social interactions individuals engage, which social conditions individuals need to practice their way of life (Bergmüller and Taborsky 2010), or how individuals specialize in their social behavior (Lucas and Sokolowskia 2009, Zimmermann et al. 2017). Therefore, a major task of the present article is to clarify how these concepts differ and relate to each other (see the “What are individualized social niches?” section).

*Social niche* is sometimes used in opposition to *ecological niche* (e.g., Flack et al. 2006) in order to stress that studies of ecological niches are focused on abiotic and heterospecific biotic environmental conditions (i.e., biotic conditions shaped by heterospecific interactions such as predator–prey and mutualistic interactions). Because environmental conditions, however, also include conspecific biotic conditions, strictly speaking, social niches are subtypes of ecological niches (on the species, population, and individual levels). This is depicted in figure 1. A more integrative terminology also highlights that, in most cases, both abiotic and biotic (including conspecific and heterospecific) environmental conditions—in particular, the social environment—will be relevant to an individual's fitness. For reasons of simplicity, however, we will use the term *social niche* (not *social ecological niche*) in the present article. Similarly, when using the term *social niche*, we refer to individualized social niches. We are aware that there might be processes that involve nonindividualized social niches, such as collective social niche construction (Sueur et al. 2019). In the present article, we focus on individualized social niches because individuals frequently differ in their social interactions, and this has important ecological-evolutionary consequences.

**Figure 1.**
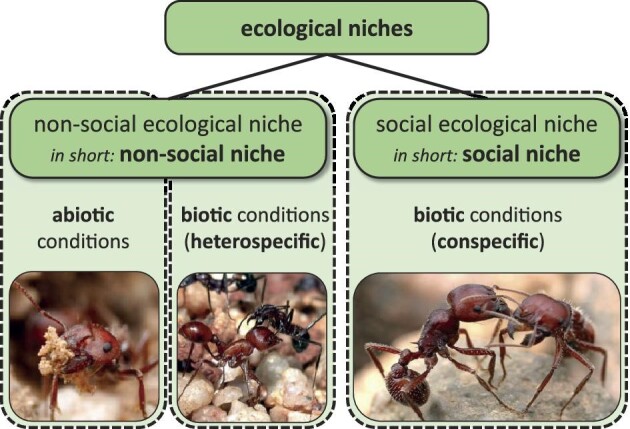
Two subtypes of ecological niches: non-social niches and social niches. Non-social niches include abiotic and biotic (heterospecific) conditions, whereas we assume that social niches include only biotic (conspecific) conditions. The pictures from left to right: Individual harvester ants (***Pogonomyrmex barbatus***) differ in their non-social niches—for instance, where and how they excavate a nest and how they interact with individuals of other ant species (***Pogonomyrmex californicus***; Johnson 1992, 2001). They also differ in their social niches, for instance how they socially interact with conspecifics, such as nestmates or nonnestmates, individuals from different castes, or individuals at different developmental stages (Clark et al. 2006, Smith et al. 2008). Photographs: Alex Wild, https://myrmecos.net.

Although it is a simplification to restrict social niches to biotic environmental conditions influenced by conspecific interactions, it makes sense for the purposes of the present article, because only conspecifics show reproductive competition. Heterospecific interactions can be very important—for example, in the case of pathogens or parasites-but they typically affect an individual through reduction of its survival or reproductive value. In contrast, other authors include all interactions with conspecifics and heterospecifics in the social niche of an animal (e.g., Ryan 2011).

Our goal in the present article is to further clarify what individualized social niches are and how they originate and change. We follow Saltz and colleagues (2016) in their call for a clear and unifying analysis of the concept of a social niche. Even though their paper has brought a lot of conceptual clarity to the debate, we think that there are still some open conceptual questions that need to be addressed. To understand what an individualized social niche is, we need to know what it consists of, how it differs from other socioecological entities (such as social environments, social interactions, social roles, but also ecosystems and environments in general), and what determines the boundaries of social niches—that is, what belongs to a social niche and what does not (see the “What are individualized social niches?” section).

We then uncover three different processes that influence the development and change of individualized social niches: social niche choice, social niche conformance, and social niche construction (see the “How individualized social niches originate and change” section). Niche choice, niche conformance, and niche construction (NC^3^) are also referred to as *mechanisms* (https://doi.org/10.32942/osf.io/7h5xq [preprint: not peer reviewed], Trappes et al. 2022, Kaiser and Trappes 2023), but we prefer the term *processes* (as used in Müller et al. 2020, Müller and Junker 2022) to distinguish them clearly from, for instance, hormonal, genetic, or epigenetic mechanisms underlying individual differences. Our central claim is that NC^3^ processes explain how individualized social niches originate, thereby specifying and extending the process that has been termed *social niche specialization* (e.g., Bergmüller and Taborsky 2010, Montiglio et al. 2013, von Merten et al. 2017, McCune et al. 2018). In line with how Müller and colleagues (2020) and Trappes and colleagues (2022) defined NC^3^ processes in general, we define social niche choice as the process by which a focal individual selects a certain social environment. Social niche conformance is the process by which a focal individual adjusts its social behavior in response to the social environment, and social niche construction is the process by which a focal individual makes changes to its social environment. All three processes lead to a change in the phenotype–environment match and in the individual's inclusive fitness, which modulates the individualized niche (Kaiser and Trappes 2023). While some theoretical work has been performed on social niche construction, social niche choice and conformance have not been recognized as independent niche-altering processes but, rather, have been ignored or lumped together with social niche construction (e.g., Saltz et al. 2016, Stanley et al. 2018, Mielke et al. 2021). We argue that recognizing the differences among these three processes will enhance and stimulate empirical research on social niches.

Our approach in the present article is integrative in at least two ways. First, it brings together empirical studies of social niches with a philosophical analysis of the concept of social niches and how they originate and change. To achieve this goal, we make use of philosophical resources to contribute to the existing theoretical discussion. We argue that theoretical clarifications about the concept promote empirical studies of social niches. Second, our approach integrates studies of behavior, ecology, and evolution, bringing together the different perspectives on social niches of these disciplines. We apply the framework of NC^3^ processes, which has been developed for individualized ecological niches in general (Müller et al. 2020, Trappes 2021, Trappes et al. 2022, Kaiser and Trappes 2023), to the social context. Furthermore, by defining social niches as subtypes of ecological niches, rather than something different, we generate an integrated niche concept.

## What are individualized social niches?

The study of social niches, of their underlying mechanisms, and of their ecological and evolutionary consequences is gaining increasing prominence in the field. The concept of a social niche, however, is still used in a variety of different senses. Social niche is specified, for instance, in terms of the social conditions that an individual needs to practice its way of life, in terms of the social roles an individual plays, in terms of the social interactions (i.e., interactions with conspecifics) that an individual engages in, in terms of the social environment, in terms of social situations that individuals are typically found in, in terms of social networks or patterns of social interactions, in terms of social groups that individuals are members of, in terms of social behaviors of individuals, and in terms of social selection pressures (for references, see table 1). Many researchers refer to more than one of these concepts to explain what social niches are. References to social interactions, social environments, and social roles are most frequent.

**Table 1.** Different concepts used by other authors to specify social niches.


**Social niche is specified in terms of…**	**References**

Social conditions	Bergmüller and Taborsky 2010, Bar Ziv et al. 2016, von Merten et al. 2017
Social roles	Bergmüller and Taborsky 2010, Montiglio et al. 2013, McCune et al. 2018; less prominently in von Merten et al. 2017
Social interactions	Bergmüller and Taborsky 2010, Kohn et al. 2011, Ryan 2011, Fisher et al. 2016, Mielke et al. 2021, Mutwill et al. 2020; less prominently in Flack et al. 2006, Montiglio et al. 2013
Social environment	Formica and Tuttle 2009, Ryan 2011, Saltz et al. 2016, Fisher et al. 2016, Mielke et al. 2021; less prominently in Bergmüller and Taborsky 2010, Bar Ziv et al. 2016, Stanley et al. 2018
Social situations	Montiglio et al. 2013, Mutwill et al. 2020
Social networks or patterns of social interactions	Flack et al. 2006, Fisher et al. 2016, Krause et al. 2009, Sueur et al. 2019
Social groups	McCune et al. 2018
Social behaviors	Montiglio et al. 2013, Mielke et al. 2021
Social selection pressures	Lipatov et al. 2011

In their influential review, Saltz and colleagues (2016) recognized this variety of terms and understandings and provide an explicit definition of *social niche* that aims at unifying the different ideas present in current social niche research. They define the social niche on the individual level as “the set of social environments in which the focal individual has nonzero inclusive fitness” (Saltz et al. 2016, p. 351). In accordance with this definition, they identify social environments and fitness as the two key elements of (individualized) social niches and argue that changes in social niches (e.g., through social niche construction) require changes either in the social environments or in fitness (Saltz et al. 2016, p. 356).

The analysis by Saltz and colleagues (2016) represents an important contribution to clarifying and unifying the concept of a social niche. Our approach is similar to theirs in that it focuses on individualized social niches and regards fitness as the essential qualifier of the social niche of an individual (in the present article, by *fitness*, we mean inclusive fitness without always making it explicit). However, we think that there is still theoretical work to be done on the concept. In particular, it must be clarified how the different terms involved in specifying the concept of a social niche, such as *social interactions, social environment*, and *social role*, relate to the social niche concept and to each other. Clarifying these conceptual relations highlights, for instance, that social niches are distinct from social environments and that these terms should not be used synonymously or interchangeably—contrary to what is often done in studies of social niches. Our analysis also explains why social niches do not exist independently of individuals, and it emphasizes the importance of social interactions for social niches.

### Novel definition of the social niche

**Figure 2.**
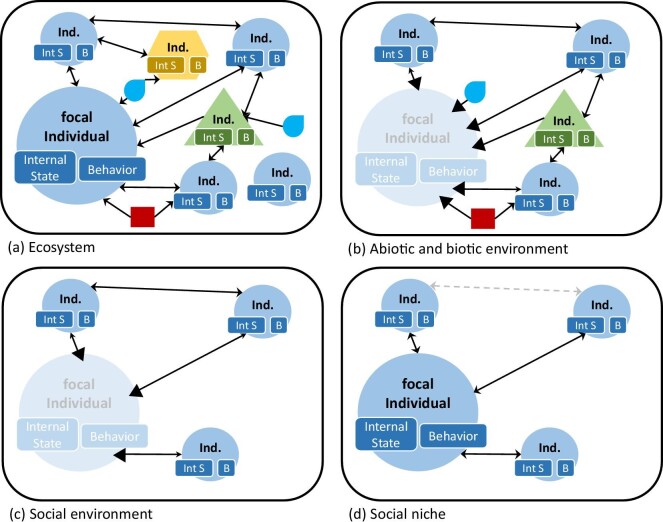
Schematic depictions of differences between a focal individual's ecosystem (a), abiotic and biotic environment (b), social environment (c), and social niche (d). Individuals (Ind.) of the same species as the focal individual are represented by circles, heterospecific individuals by triangles or hexagons. Individuals possess internal states (Int S), such as hormonal, physiological or immunological states, and they exhibit behaviors (B), such as mating, aggressive behavior or foraging behavior. The rectangles and drops represent abiotic conditions. The arrows represent interactions (bidirectional or unidirectional causal relations). The heads of the arrows leading to the focal individual are highlighted in panels (b) and (c), which express that the effects on the focal individual are most important. The dashed arrow (d) represents a social interaction between nonfocal individuals that indirectly influences the focal individual but is not in the main focus of the social niche of this focal individual. The blurred circles indicate that the focal individual, although present, does not constitute a part of the represented entity (e.g., in the case of environments).

We define the concept of a social niche as follows: “An individualized social niche is the unit consisting of a focal individual and only those social interactions with other conspecific individuals that influence the focal individual's inclusive fitness.” On the basis of this definition, we can further specify what social niches are by distinguishing them from other ecosocial entities, such as ecosystems, environments in general, and social environments. Figure 2 illustrates this difference.

In our definitions, an *ecosystem* (figure 2a) includes all individuals of all species within a defined area and its abiotic conditions, as well as the various interactions among all of these components. The *abiotic and biotic environment* (figure 2b) of a focal individual contains only those abiotic factors (e.g., water availability, temperature) and those individuals (of the same and other species) of the ecosystem that directly affect the focal individual, as well as the interactions among these individuals and abiotic factors. The *social environment* (figure 2c) focuses on the conspecifics and how they interact with and affect the focal individual. The social environment also includes all interactions among nonfocal individuals. Both environments (abiotic or biotic environment and social environment) are external to the focal individual and therefore exclude the focal individual itself (represented by the lighter blue colors) but include the interactions between the focal individual and other individuals and abiotic factors. Because environments affect individuals, those sides of the interactions leading to the focal individual are emphasized (by thicker black arrowheads).

Finally, the *social niche* (figure 2d) consists of the focal individual and its social interactions with other conspecific individuals that directly affect the focal individual's inclusive fitness. The focus is on direct interactions with the focal individual. There are also indirect effects; for instance, the interaction between two nonfocal individuals (i.e., conspecifics) could influence a focal individual's social interactions and therefore its social niche (represented by the gray dashed arrow in figure 2d). For example, the fighting of two dominant animals may enable the focal individual to mate. These indirect effects are sometimes important for understanding a certain social process or phenomenon and will therefore be included in empirical studies. However, empirical studies often concern different individualized social niches and are not limited to the perspective of a single focal individual. We argue that when studying the social niche of a specific focal individual, the social interactions that this focal individual is involved in should be the main focus. Indirect effects, such as interactions between conspecifics that may also affect the focal individual, certainly take place but are not in the focus when considering the social niche of this specific focal individual.

Figure 2 also represents that there are three major differences between an individual's social environment and its social niche. First, social environments encompass interactions between all individuals, whereas social niches include only those social interactions between individuals that affect the focal individual's inclusive fitness. In addition, social niches focus on direct social interactions with the focal individual. Second, the social environment is external to the focal individual and affects it. This implies that social environments involve social interactions but emphasize one side of them—namely, that the social environment (i.e., the conspecifics) affect the focal individual. Third, the social environment surrounds the focal individual but the focal individual is not a part of the social environment. By contrast, focal individuals are integral parts of social niches; a social niche encompasses the focal individual. Thinking about social niches implies thinking about focal individuals and their fitness-relevant interactions. Because the individual needs to perceive its social interaction partners and integrate the signals into its cognitive functions and decision making, it must also be part of the social niche. For example, during courtship, the individual is driven by its physiological state and uses its communication systems to interact with conspecifics (Ryan 2011), which will ultimately influence its fitness.

**Figure 3.**
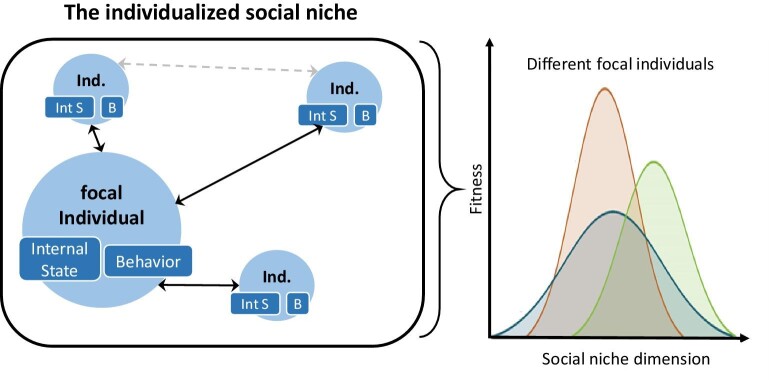
The individualized social niche. Individualized social niches consist of the focal individual and those social interactions that affect the focal individual's inclusive fitness (often measured in terms of fitness proxies). Social interactions can be mediated through behaviors or internal states of individuals. Different focal individuals have different individualized social niches that result in individual-specific fitness functions. The focal individual in the left part has the fitness function with the lowest maximum fitness value in the right part. The other two focal individuals with the other two fitness functions are not included in the left part of the figure. For abbreviations see figure 2.

Figure 3 illustrates our understanding of the (individualized) social niche and highlights the central role of the focal individual and its social interactions as well as the importance of fitness for determining what belongs to the social niche. The right part of figure 3 shows that different focal individuals can have different individualized social niches that result in individual-specific fitness functions for certain social niche dimensions (https://doi.org/10.32942/osf.io/7h5xq [preprint: not peer reviewed]).

### Social niches and social interactions

Our understanding of the social niche deviates from the definition by Saltz and colleagues (2016) in two respects. First, we argue that social niches are made up of social interactions, not of social environments, and second, we require the social interactions to (positively or negatively) influence an individual's fitness, rather than requiring that the individual needs to have nonzero fitness. We consider the second difference as rather minor and focus on explaining the first difference in this section.

We think that social interactions are central to understanding social niches. Social interactions are the entities that compose a social niche—together with the focal individual and the other individuals that the focal individual interacts with. This does not mean that the social environment of an individual is irrelevant to its social niche or that there is no overlap between social niches and social environments. Social interactions make up social niches, and they make up or are part of social environments. However, not every social interaction that is part of the social environment is also part of the social niche. There are two crucial differences between social environments and social niches. First, individualized social niches are social niches of focal individuals. They draw our attention to the social interactions with this focal individual and place interactions between other conspecifics into the background. One could therefore say that social niches primarily consist of social interactions with the focal individual, whereas social environments also include social interactions between conspecifics that may indirectly affect the focal individual (see the difference between figure 2c, 2d). Second, only social interactions that influence the focal individual's fitness—not fitness-neutral interactions, such as animals sniffing at each other—are part of the social niche. For example, females and males of the parasitoid wasp *Nasonia giraulti* mate almost exclusively within the pupal shell of their fly hosts (Trienens et al. 2021). Therefore, the social niche of each individual within a host is limited to the individuals present. Social interactions or environments (e.g., sex ratio) in other hosts are not relevant for reproductive behavior of the focal individuals. Because those other conspecifics do not influence the fitness of the focal mating individuals, they are not part of their social niche.

We also think that it is important to emphasize that the focal individual is part of the social niche but not part of the social environment. Conceiving of a social niche as a “set of social environments,” as Saltz and colleagues (2016, p. 351) did, blurs the difference between an individual's social niche and its social environment. Social niches do not consist of several complete social environments. Rather, they consist of the focal individual plus certain parts of the focal individual's unique social environment—namely, those social interactions with the focal individual that affect its fitness. For example, adult sawflies of the species *Athalia rosae* can acquire certain chemical compounds either directly by nibbling on the plant *Ajuga reptans* (i.e., interaction with the biotic environment) or by nibbling on a conspecific that had access to this plant. Having these compounds increases their mating success (Amano et al. 1999), as well as their defense against predators (Singh et al. 2022), highlighting the importance of chemical communication in social niche individualization processes (Müller et al. 2020). Thereby, individuals are pursuing different social interactions, in which they may either mate or fight to acquire these compounds from a conspecific (Paul and Müller 2022). For the focal individual, only its contacts with other individuals (with or without these compounds) are relevant and will affect its fitness. Mating or fighting among other conspecifics may indirectly affect its own chances or opportunities of contacts but is not directly part of its individualized social niche. At least, we prioritize the direct effects because only those can be directly investigated.

**Figure 4.**
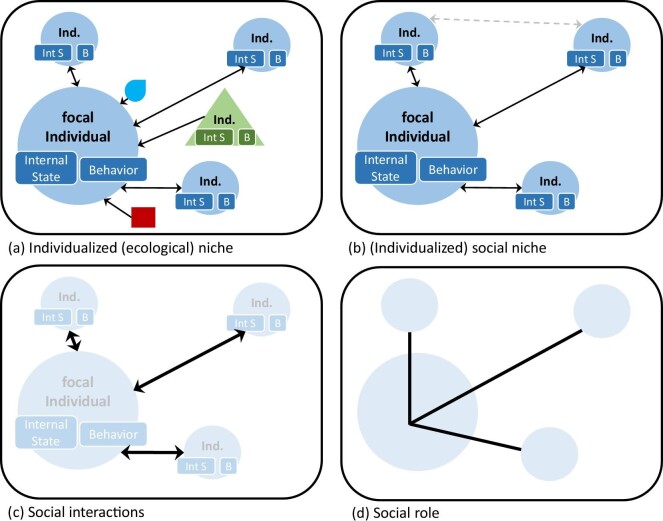
The difference between a focal individual's ecological niche in general (a), its social niche (b), which is the same as figure 2d, its social interactions (c), and one of the social roles that a focal individual performs (d). Individuals (Ind.) of the same species as the focal individual are represented by circles, heterospecific individuals by triangles. Individuals possess internal states (Int S), such as hormonal, physiological or immunological states, and they exhibit behaviors (B), such as mating, aggression or foraging. The rectangle and drop represent abiotic conditions. The arrows represent interactions (bidirectional or unidirectional causal relations). The blurred circles indicate that individuals, although present, do not constitute a part of the represented entity (e.g., in the case of social interactions and the social role).

How should the concept of social interaction be understood? Social interactions are causal relations that are often but not necessarily bidirectional; that is, the individuals influence each other (represented by the double-headed arrows in figure 3 and in figure 4a–4c). In monogamous species, for example, males and females can form strong social pair bonds. To establish and maintain such bonds, bidirectional social interactions, such as affiliative behaviors or vocalizations such as duetting, are often very important (Geissmann and Orgeldinger 2000, Pultorak et al. 2018). Their frequent bidirectionality contrasts social interactions with interactions between individuals and abiotic factors that are commonly unidirectional, leading from the abiotic factor to the individual (except for cases of niche construction).

In many cases, one individual behaving in a certain way will influence the behavior of another individual (e.g., the focal individual approaching another individual causes the latter to avoid or approach the focal individual). It is, however, important not to reduce social interactions to behavioral interactions. Social interactions can be mediated both by behaviors and by the internal states of individuals. For example, male sex pheromones, crucial for chemical communication, are often released during courtship behavior to elicit copulation, but this works only if the female is still receptive (e.g., van den Assem et al. 1980). Figures 2–4 clearly depict this. Accordingly, we define a social interaction as the influence of the behavior and internal state of a conspecific on the behavior and internal state of the focal individual and vice versa. This causal influence on the behavior and internal state of the interacting partners can, but does not need to, also influence the focal individual's fitness. Individuals in a group interact with each other frequently, but many interactions are not relevant to fitness. For example, young male golden snub-nosed monkeys (*Rhinopithecus roxellana*) often live in male groups before they are able to establish a harem. In such all-male groups, sexual behavior between males occurs frequently, but it is not relevant to fitness for the focal individual (Fang et al. 2018). Contrary to social environments, social niches consist only of fitness-relevant social interactions. This is important to emphasize because we should not build the requirement of a fitness effect decisive for social niches already into the concept of social interactions.

### Social roles

Another concept that is frequently used to explain what social niches are is the social role. Social roles are often conceived of as being distinct parts of a social network. Individuals can play more than one social role at the same time, such as being a dominant female, being a mating partner, and being a mother (motherhood). These social roles constrain and allow for certain social interactions (e.g., threatening or fighting, mating, nursing), as well as related social behaviors (e.g., approaching or showing aggression, mating behavior, nursing behavior). Social roles are therefore not the same as social interactions. A social role, such as motherhood, typically involves more than one social interaction that can be targeted against different conspecifics (e.g., nursing, giving birth, protection, warding off competitors). Therefore, the social role depicted in figure 4d consists of more than one line.

Moreover, the concept of a social role and the concept of a social interaction highlight different aspects and present different perspectives when studying social niches (figure 4c, 4d). Whereas the concept of social roles highlights the structure of social groups and the roles that individuals can play in social networks, the concept of social interactions emphasizes causal processes and how individuals influence and change each other’s social niche according to our definition. In figure 4, we therefore use lines to represent social roles and arrows to represent social interactions. It should be noted, however, that this is not a fundamental difference but one of emphasis. Of course, social networks are built on the basis of social interactions between individuals, and filling out a social role also requires dynamic causal processes.

In general, the concept of a social role is useful in regard to species or organisms that have larger social groups that go beyond a single family. The concept of a social niche is therefore more encompassing than the concept of a social role, both in terms of types of interactions that it refers to and number of species that it can be applied to.

### Core theoretical assumptions and empirical relevance

In the preceding section, we developed four core theoretical assumptions concerning social niches (see also box 1). Our definition says that social niches consist of the focal individual, its conspecifics, and those social interactions (between focal individual and conspecifics) that affect the individual's fitness. This emphasizes, first, that focal individuals do not occupy social niches but are integral parts of them; that is, they *realize* a certain social niche. This implies that a social niche is not a space that exists per se but can only be realized with the individual itself. Second, our definition recognizes two different types of niche elements that are characteristic of social niches: conspecific individuals and social interactions primarily with the focal individual. Third, individualized social niches consist of only those social interactions that fulfill two conditions: they primarily take place between the focal individual and its conspecifics, and they affect the fitness of the focal individual. Fourth, our definition allows us to clearly distinguish social niches from social environments. An individual's social environment excludes the focal individual, whereas the social niche includes the focal individual. In addition, social environments consist of all kinds of social interactions between the individuals, whereas a social niche consists of only those social interactions between a focal individual and its conspecifics that affect its fitness.

These core theoretical assumptions clarify the concept of a social niche and its relation to other similar concepts. Therefore, they also contribute to promote empirical research on social niches. For instance, the third theoretical assumption simplifies measurements of social niche dimensions because it provides guidance to study primarily those social interactions with the focal individual that directly affect the focal individual's fitness. Given the complexity of indirect effects on an individual's fitness, this helps to identify the most relevant dimensions that are part—or modulate—the individualized social niche. Moreover, the third theoretical assumption implies that empirical studies must include measurements of fitness proxies when studying social niches (figure 3). Another example of the empirical relevance of

Box 1. **Definition and core theoretical assumptions about social niches.**

Definition

An individualized *social niche* is the unit consisting of a focal individual and only those social interactions with other conspecific individuals that influence the focal individual's inclusive fitness.

**Core theoretical assumptions**

(1) A *focal individual* is an integral part of its social niche. A social niche can only be realized with the focal individual.

(2) Two types of *niche elements* are characteristic of social niches: conspecific individuals and social interactions (primarily with the focal individual).

(3) Social niches consist of only those *social interactions* that fulfill two conditions: first, they primarily take place between the focal individual and its conspecific(s), and second, they affect the fitness of the focal individual.

(4) Social niches differ from *social environments*: first, niches include the focal individual while environments exclude it, and second, environments include fitness-neutral social interactions while niches only include fitness-relevant social interactions.

theory is that the first, second, and fourth theoretical assumptions contribute to developing a conclusive study design. For instance, the second assumption implies that we must study social niches by measuring the behaviors, as well as the internal states of the socially interacting individuals. The fourth assumption reveals that one should not confuse social environments and social niches: whether one wants to study a social environment or a social niche (and even more specifically, the social niche of which focal individual one wants to study) will considerably influence which social interactions and which behaviors and internal states of which individuals one should measure.

## How individualized social niches originate and change

Within populations, individuals differ in their social niches. They show different social behaviors, and these behavioral differences can be stable over time and consistent over contexts. This is referred to as *animal personality* (e.g., Montiglio et al. 2013, Sih et al. 2015, von Merten et al. 2017, Kaiser and Müller 2021). Individuals engage in different social interactions and occupy different, more or less stable social roles (McCune et al. 2018). The phenomenon that individuals specialize in their social niches—that is, develop (more) *individualized* social niches—is often referred to as *social niche specialization* (e.g., Bergmüller and Taborsky 2010, Montiglio et al. 2013, von Merten et al. 2017, McCune et al. 2018). Reference to social niches is supposed to explain how stable and consistent individual differences in social behavior and internal states arise and change (Montiglio et al. 2013, von Merten et al. 2017). This claim, however, raises the question of how the social niche concept can provide explanatory power. In other words, how can thinking about niches help to explain how individual differences in social behavior (and internal states) arise and change?

We think that our social niche concept is helpful because it brings together the different fields of behavioral biology, ecology, and evolutionary biology to study the origin and changes of social behavioral variation and personality in a coherent, explanatory framework. In particular, the niche concept allows us to distinguish three different types of niche-altering processes that bring about individual differences in social behavior (and internal states): the process of niche construction, the process of niche conformance, and the process of niche choice (together referred to as *NC^3^*). The framework of NC^3^ processes has been developed for individualized ecological niches in animals (https://doi.org/10.32942/osf.io/7h5xq [preprint: not peer reviwed], Müller et al. 2020, Trappes et al. 2022, Kaiser and Trappes 2023) and recently also aligned with plants (Müller and Junker 2022). In the present article, we apply and adapt this framework specifically to individualized social niches. The framework of NC^3^ processes is fruitful for studying social niches because it specifies *how* individualized social niches arise and change over time.

### Social niche construction

If authors discuss more specific processes of social niche specialization (or individualization), they typically focus on niche construction. Many authors apply niche construction theory and think of social niches more in terms of selection pressures, or they operate with a quite broad notion of niche construction that encompasses niche choice and niche conformance (e.g., Saltz et al. 2016, Stanley et al. 2018, Mielke et al. 2021). A broad notion of niche construction, however, has the disadvantage that it obscures important differences between distinct proximate processes of how individuals generate or change their individualized social niches.

We therefore adopt a narrow notion of niche construction, according to which a focal individual constructs its social niche by actively making changes to its social environment in a way that affects its fitness (Trappes et al. 2022). A first requirement of social niche construction is that the individual directly changes the way conspecifics behave or more generally influence the composition or dynamics of its social environment. For example, recently, it has been shown that multiple individual and relationship characteristics (such as rank, kinship, and affiliative relationships) influence decisions about interventions in grooming of female rhesus macaques (*Macaca mulatta*). Rather than simply gathering information, rhesus macaques manipulate interactions between others to their own benefits and construct their own social niche (Mielke et al. 2021, see figure 6a). Our narrow notion of niche construction allows us to distinguish social niche construction from two other processes that give rise to or change individualized social niches: social niche conformance and social niche choice. Social niche construction does not lead to a change in the social behavior of the focal individual itself (which would be social niche conformance), even though it can cause a subsequent change in the social behavior of the focal individual as a reaction to or feedback of the construction process. Social niche construction also does not consist of the focal individual selecting a different social environment—for example, by relocating or choosing a different partner (which would be social niche choice). In the case of social niche construction, through its behavior or internal state the focal individual actively changes the social behavior of interacting conspecifics.

A second requirement of social niche construction (that it shares with choice and conformance) is that the focal individual actively changing its social environment must result in a change of the focal individual's phenotype–environment match, fitness, and consequently its individualized social niche (Trappes et al. 2022, Kaiser and Trappes 2023). Saltz and colleagues (2016, p. 356) argued that niche construction does not necessarily involve a change in the focal individual's fitness. Contrary to this claim, we think that all cases of (social) niche construction (as well as of niche choice and conformance) must affect the individual's fitness. A mere change in the social environment that does not affect the focal individual's fitness is not a case of niche construction because the social niche does not change. These cases might be rare (and impossible to detect empirically) because most changes of an individual's social environment will at least slightly change the individual's fitness. Still, these cases are theoretically possible, and if they existed, they would not be cases of niche construction.

Non-social niche construction also requires that it has consequences for other individuals of the same or other species. The classic example is that of the beaver, which by actively building its dam affects the nutrient cycle, water flow, and therefore the composition of plant communities (Nica et al. 2022). However, in cases of non-social niche construction the effects on the fitness of other individuals is often ignored.

### Social niche conformance

Niche conformance is defined as the process by which an individual adjusts its phenotype in response to the environment (Müller et al. 2020, Trappes et al. 2022). Accordingly, social niche conformance means that individuals change their phenotype in response to the social environment and social interactions. What makes niche conformance social is the environment being social, not the adjusted behavior necessarily being social. For example, a worker in a honey bee (*Apis mellifera*) colony reacts to its social environment (e.g., absence or presence of a queen) with physiological and behavioral changes (e.g., develop their ovaries and lay eggs or suppress these processes if a queen is present), which are changes in individual behavior and internal states, respectively, but not changes in social behavior (Wyatt 2003). Likewise, male guinea pigs (*Cavia apera* f. *procellus*) living in different social environments (high or low individual number) during adolescence show different neuroendocrine responses. These lead to an adaptive shaping of their behavioral phenotypes; that is, individuals show high or low aggressiveness (see figure 6b). In this way, they conform to the respective social situation and use either resource defense or queuing strategies, realizing different individualized social niches (Sachser et al. 2018). Interestingly, such social niche conformance processes can even be observed at the level of an animal's personality. For example, it was shown that Gouldian finches (*Chloebia gouldiae*) adjust their behavior according to the personality of their partners: where a bird's partner is bolder, the focal individual becomes bolder; where a bird's partner is shyer, the focal individual becomes shyer, pointing toward an adaptive shaping of the personality type (King et al. 2015).

Saltz and colleagues (2016) argued that the difference between social niche construction and social niche conformance (what they referred to as *phenotypic plasticity*) is merely a matter of perspective. The focal individual adjusting its phenotype in response to the social environment (social niche conformance) is, from the perspective of the conspecifics, a case of social niche construction because it is the social behavior of the conspecific that leads to the change of the social behavior of the focal individual. In turn, when conspecifics adjust their phenotypes in response to the social environment (social niche conformance), from the perspective of the focal individual we have a case of social niche construction. This is one reason Saltz and colleagues (2016) grouped phenotypic plasticity under the label of social niche construction.

**Figure 5.**
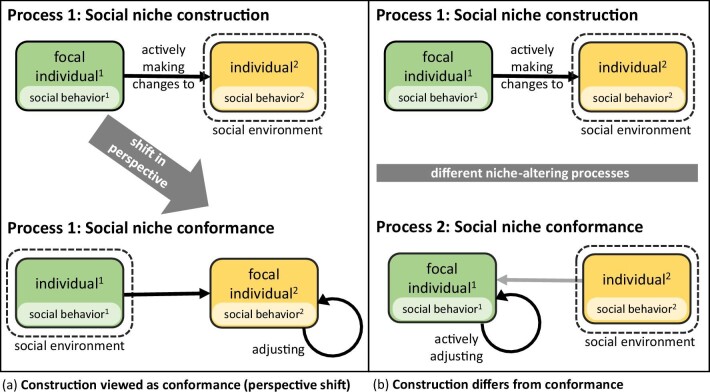
The difference between social niche construction and conformance. If a process of social niche construction is viewed from the perspective of the conspecific (shift in perspective), it is a process of social niche conformance because the focus is on the focal individual adjusting its social behavior in response to the social behavior of individual (a). By contrast, if the perspective—that is, the focal individual—remains the same, the processes of social niche construction and conformance can be clearly distinguished (b).

We agree that such a shift in perspective is possible and that from the perspective of the involved conspecifics a process of social niche construction is a process of social niche conformance (see figure 5a). However, construction and conformance are nevertheless different types of niche-altering processes, which involve different individual–environment interactions and in which the focal individual plays a different role (see figure 5b): in social niche construction, the focal individual actively changes the conspecifics’ social behavior, whereas the conspecifics may be passive or merely responding. In social niche conformance, by contrast, the focal individual actively changes its own social behavior in response to the social behavior of its conspecifics.

Box 2. **Three processes giving rise to or changing individualized social niches: social niche construction, conformance and choice (social NC^3^ processes).**

Main characteristics

(see also Kaiser and Trappes 2023; Trappes et al. 2022)

(1) Social NC^3^ processes give rise to or *change individualized social niches* (i.e., they are niche-altering processes).

(2) Social NC^3^ processes result in: first, a *change in the match* between the focal individual's social behavior and its social environment, second, a *change in inclusive fitness* of the focal individual.

(3) The focal individual has an *active role* in NC^3^ processes.

**Social niche construction**

Social niche construction is the process by which a focal individual *makes changes to its social environment* (i.e., to the social behavior of its conspecifics).

**Social niche conformance**

Social niche conformance is the process by which a focal individual *adjusts its behavior or internal state* in response to the social environment (i.e., social behavior and internal states of conspecifics).

**Social niche choice**

Social niche choice is the process by which a focal individual *selects a different social environment* by relocating or selecting other conspecifics to interact with.

Even though such a shift in perspective (depicted also in figure 5a) is theoretically possible, in practice, the choice of the focal individual is a deliberate decision made on empirical grounds. When studying a specific process or phenomenon, we cannot simply change perspective and select a different focal individual, because this will change the process or phenomenon that we study, and it will change social interactions that we need to measure (because different individuals have different individualized social niches).

For example, in foundress associations of the wasp *Polistes gallicus*, all individuals have the same reproductive potential, but one individual becomes dominant and the principal egg layer. Females who want to join foundress associations can either challenge the dominant queen and become the dominant individual themselves (social niche construction) or become subdominant and change their behavior (predominantly foraging or brood care) and internal status (reduced ovary activity) to conform to the existing social environment (social niche conformance; West-Eberhard 1969, Röseler et al. 1985). Individuals can choose either strategy but both are fundamentally different social niches and come about by different processes. In our view, it is preferable to keep the two strategies apart and not to view the process of social niche conformance (becoming a worker) also as a case of social niche construction. In this example, researchers want to understand the process of an individual wasp joining an existing foundress association and choosing the strategy of becoming a worker by adjusting its social behavior to the existing social environment. This process cannot be viewed as a case of niche construction because, first, for understanding and studying this process, the focal individual needs to be the individual adjusting its strategy to become a worker, not one of its many conspecifics, and second, the conspecifics do not have an active role in this process, they are described as constituting the existing social environment that the focal individual actively responds to.

One might further object that a focal individual changing the social behavior of its conspecifics (social niche construction) often feeds back and causes a change also of the social behavior of the focal individual (social niche conformance). This is true and such feedback loops play an important role in many complex social behaviors (e.g., Couzin 2009, Wild et al. 2021). If these processes happen sequentially and quickly, one after another, it might become difficult to empirically distinguish them. However, conceptually, these two processes are not the same and should be kept apart, while at the same time acknowledging the importance of feedback and emphasizing the dynamics of individualized social niches that are realized in complex, socially heterogeneous networks (Krause et al. 2009).

Considering the colony founding phase of pleometrotic *Polistes* spp. species (i.e., joined colony founding of several founding individuals with the same reproductive potential), we see a more dynamic and complex picture emerging. A focal individual that becomes dominant and the sole egg layer of a colony foundress association is first engaging in social niche construction by means of aggressive interactions with her cofoundresses. Immediately afterward, the focal individual engages in social niche conformance as she changes her social behavior (no more physical aggression but pheromonal signaling), physiology (egg production), and individual behavior (no foraging or brood care). Individuals that lost in these dominance interactions will become workers conforming like the dominant individual (queen) to their social niche (deactivating their ovaries and forage, caring for their brood, or building the nest). Social interactions between those workers lead to nonreproductive division of labor and, in the end, result in a complex social network that we usually call a colony. If a dominant individual dies, is challenged by another individual, or is removed by an experimenter these processes will be repeated (e.g., Field and Leadbeater 2016). In addition, as colonies grow in size, the mechanisms of social dominance may switch from physical interactions to pheromonal communication (e.g., Sledge et al. 2001). This example highlights the huge dynamics that occur in niche realization processes.

### Social niche choice

The third NC^3^ process is niche choice, which is understood as the process by which a focal individual selects a different environment to interact with (Müller et al. 2020, Trappes et al. 2022). Individuals select a different social environment for instance by relocating or choosing other conspecifics to interact with. For example, focal individuals with different personalities may differ in their choice of social interaction partners. In Trinidadian guppies (*Poecilia reticulata*) it has been observed that contact patterns are strongly influenced by personality, with individuals with different personality types preferring different social interaction partners (Croft et al. 2009). Likewise, a study on chimpanzees (*Pan troglodytes*) showed that similar to humans, chimpanzees’ friendships are partly determined by similarities in personality, particularly with respect to those characteristics that are relevant for socially positive and cooperative behavior (Massen and Koski 2014). This selection of a different social environment often improves the match between the focal individual's social behavior and its social environment (i.e., the social behaviors of the conspecifics) and enhances the fitness of the focal individual.

**Figure 6.**
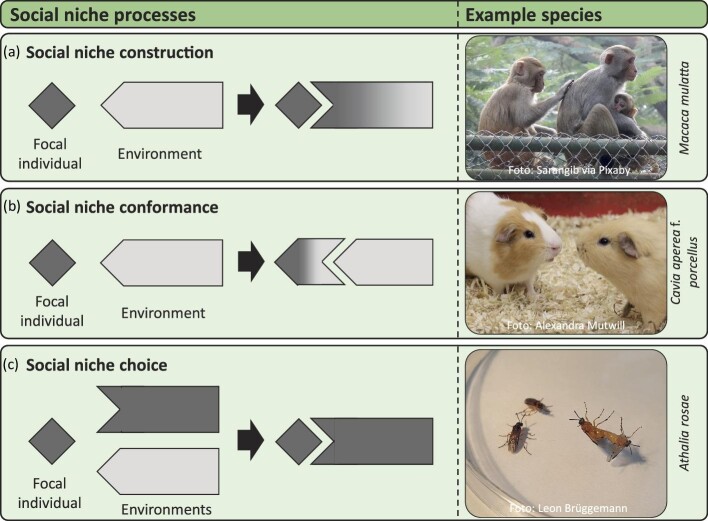
Schematic representation of the social NC^3^ processes and examples. The focal individual is represented as a dark gray square, the social environments as rectangles with white or dark grey color and different shapes. In social niche construction (a), the social environments change its shape and color in a way that matches the focal individual better, representing that the focal individual actively changes its social environment. In social niche conformance (b), the color and shape of the focal individual changes in a way that matches the social environment better, representing that the focal individual adjusts its social behavior. In social niche choice (c), the focal individual chooses the social environments that have the same color and whose form fits best. Examples are depicted on the right. See the text for details.

Another example of social niche choice is the mate choice of *Athalia rosae* sawflies (see figure 6c). Females without clerodanoid access may either choose a male mate with or without clerodanoid access, making themselves either also more attractive for social interactions via nibbling or not (Paul and Müller 2022). Overson and colleagues (2014) showed that if queens from a haplometrotic or pleometrotic population of harvester ants got a choice to found a nest alone (haplometrotic) or with other queens (pleometrotic) they all choose the latter, although, in groups with haplometrotic queens, all but one queen were killed because of aggressive interactions, whereas in groups with pleometrotic queens only, all of the queens survived and produced more offspring than the single queen nests did.

To summarize, social niche choice is different from social niche conformance in that during the choice process the social behavior of the focal individual remains unchanged. Social niche choice is different from social niche construction in that the focal individual does not actively change its social environment but merely chooses a different social environment to interact with, neither changing the old nor the new social environment. Box 2 summarizes the main characteristics that all three niche-altering processes—social niche construction, conformance, and choice—have in common and provides clear definitions of each of these processes. Figure 6 provides schematic illustrations and examples of the social NC^3^ processes.

### Empirical challenges of studying social NC^3^ processes and the value of theory

Empirically studying the processes of how individualized social niches arise and change (i.e., social niche construction, conformance, and choice) can be challenging. First, researchers need to make sure that an individual is confronted with a specific social environment. This is challenging because the social environment consists of social interactions, which are dependent on the focal individual's behavior. Second, researchers need to find ways to measure whether the behavior and internal state of the focal individual, the behaviors and internal states of its conspecifics, and the interactions between them have changed. If a change has occurred, they need to prove that the change is due to changes in social interactions. For instance, did the focal individual change its behavior and thereby actively influence a conspecific (construction), or was the focal individual influenced by the conspecific and therefore changed its behavior (conformance)? The outcome may be similar in either case, and it may be tricky to identify the right causal process. Third, researchers need to measure whether and how specific individual–environment interactions affect the phenotype–environment match and the focal individual's fitness. Match is typically measured in terms of fitness (Trappes et al. 2022), but the fitness of individuals is often challenging to measure. Researchers frequently use fitness proxies, such as growth rate, body condition, or reproductive success in a certain time period, to measure fitness.

Clear concepts and theoretical assumptions do not remove these empirical challenges but can help overcome and meet the challenges. As our way of formulating the empirical challenges already shows, our concepts of social NC^3^ processes, as well as the concepts that we introduced in the “What are individualized social niches?” section, clarify what researchers should measure when studying how individual social differences arise and change. These concepts therefore contribute to improving study designs and discussions of empirical results. Distinguishing the three social NC^3^ processes makes very clear what we need to look for and measure. For example, if we expect social niche construction to happen, we should look for how the focal individual actively changes its social environment, we should not find changes of the focal individual's phenotype, and despite the changes in the social environment, there should be good reasons to assume that the focal individual stays in its social environment and does not select a completely new one. Also, an individual can experience or initiate different niche-altering processes consecutively over a short period of time. Not lumping those together and clearly separating them in distinct processes can help to better understand the different underlying proximate and ultimate mechanisms. Of course, it might be empirically challenging to disentangle the different niche-altering processes—for instance, if feedback between them occurs or if one quickly follows the other.

The concept of social NC^3^ processes provides empirical guidance also because it implies that there is a clear focal individual that has an active role in the social niche-altering process (by either making changes to the social environment, adjusting its social behavior, or selecting a different social environment). Which individual is the focal individual can be derived from the research question being studied.

## Conclusions

We clarified the concept of the social niche in the context of individualized niches and distinguished three processes—social niche construction, social niche conformance, and social niche choice (social NC^3^ processes)—that are the key processes leading to the emergence and change of an individual's social niche. We highlighted the importance of social interactions and the focal individual and expanded the social niche beyond behavioral phenotypes, adding internal states and chemical communication. The focus on social interactions as a key element of social niches makes it a discrete and quantitative measure. This view is closer to our understanding of behavior as a quantitative trait. Finally, we clarified the differences between concepts that are sometimes interchangeably used for a social niche or that cover a specific component of a social niche. By integrating social niches into the broader concept of an individualized ecological niche we hope to avoid misunderstandings and stimulate further interdisciplinary research to answer the crucial question why and how individuals differ in their social niche.

Challenges for the future development of the social niche as a useful concept in evolution, ecology, and behavior are both empirical and theoretical. Similar to the struggles of niche construction to find its place and role in evolutionary theory, social niche construction, conformance and choice need to continue to be incorporated in ecological theory (environment–phenotype, phenotypic plasticity), behavioral theory (animal personality), and evolutionary theory (fitness, social selection). In another context, this challenge has been known for a long time—for example, in the discussion about indirect genetic effects, which can be explained as environmental influences on the phenotype of an individual that can be traced back to the expression of genes in another individual of the same species (Moore et al. 1997, Wolf et al. 1998). Empirically, we have many systems in which we, in principle, could measure all relevant parameters (i.e., all relevant social interactions over an extended period of time that determine fitness differences of several focal individuals), but it is time consuming, and it may afford developing new tools to analyze and characterize all or all major types of social niches of a population or species. Finally, more theoretical and empirical research on the concept is needed to investigate, for instance, whether social niches change and evolve at a different speed from non-social niches, whether social niches are more plastic than non-social niches, how climate change affects different niche-alteration processes, and whether there are species- or population-specific patterns of social niche changes, analogous to developmental processes during the developmental changes of an individual.

## Author Biography

Marie I. Kaiser (kaiser.m@uni-bielefeld.de) is affiliated with the Department of Philosophy and Caroline Müller is affiliated with the Department of Chemical Ecology at Bielefeld University, in Bielefeld, Germany. S. Helene Richter and Sylvia Kaiser are affiliated with the Department of Behavioural Biology and Jürgen Gadau is affiliated with the Institute for Evolution and Biodiversity at the University of Münster, in Münster, Germany. All of the authors are also affiliated with the Joint Institute for Individualisation in a Changing Environment, at the University of Münster and Bielefeld University, in Münster and in Bielefeld, Germany.
